# Reframing maternal and newborn health after the Sustainable Development Goals 2030 agenda: The role of The Lancet Commission on Maternal and Newborn Health

**DOI:** 10.1002/pmf2.70349

**Published:** 2026-06-19

**Authors:** Mehreen Zaigham, Ines Nunes, Bo Jacobsson

**Affiliations:** ^1^ Obstetrics and Gynaecology, Institution of Clinical Sciences Lund Lund University Lund Sweden; ^2^ Department of Obstetrics and Gynaecology Skånes University Hospital Malmö Sweden; ^3^ Women's and Children's Department Gaia/Espinho Local Health Unit Vila Nova de Gaia Portugal; ^4^ RISE‐HEALTH University of Aveiro Aveiro Portugal; ^5^ Department of Medical Sciences, University of Aveiro Aveiro Portugal; ^6^ Department of Obstetrics and Gynecology, Institute of Clinical Science, Sahlgrenska Academy University of Gothenburg Gothenburg Sweden; ^7^ Department of Obstetrics and Gynecology Sahlgrenska University Hospital, Västra Götaland Region Gothenburg Sweden; ^8^ Department of Genetics and Bioinformatics, Domain of Health Data and Digitalization Institute of Public Health Oslo Norway

## 1 AN UNFINISHED GLOBAL HEALTH CRISIS

Maternal and newborn health remains one of the most urgent unfinished agendas in global health. Despite decades of investment and important progress, a woman still dies every 2 minutes from complications related to pregnancy or childbirth [1, 2, 3]. In 2023, an estimated 260,000 women died from maternal causes globally, while approximately 2.3 million newborns died during their first month of life in 2022 [1, 2]. This translates into nearly 700 maternal deaths and more than 6000 newborn deaths every single day. These deaths are not only personal tragedies. They have profound consequences for families, communities, and societies, affecting children's survival, economic stability, and long‐term social development. Most importantly, most of these deaths are preventable.

Yet progress toward the Sustainable Development Goals (SDGs), adopted by all United Nations Member States in 2015 as a shared framework for improving health and development by 2030, has slowed dramatically [4]. Within SDG 3, maternal and newborn survival remains a central target, but the global maternal mortality ratio continues to remain far above the target of fewer than 70 deaths per 100,000 live births. With 2030 approaching and many targets increasingly unlikely to be met, the global health community faces a critical moment: not only to reassess what is being done, but also to reconsider how priorities are defined and whether current approaches are truly reaching the women and families most in need.

Against this backdrop, The Lancet Commission on Maternal and Newborn Health was launched in 2025 as a multidisciplinary global initiative bringing together researchers, clinicians, policymakers, advocates, and representatives from international organizations [4]. Lancet Commissions are independent scientific collaborations convened by *The Lancet* to address major global health and societal challenges through evidence synthesis, expert consensus, and policy analysis. Their purpose is not only to generate scientific knowledge but also to shape global agendas and stimulate transformative action.

The Commission on Maternal and Newborn Health aims to define priorities for the post‐2030 era and identify strategies capable of accelerating progress toward survival, dignity, equity, and quality of care for women and newborns worldwide. Importantly, the Commission also seeks to ensure that future recommendations are grounded not only in scientific evidence but also in the realities facing health systems, healthcare professionals, women, and families across diverse settings.

## 2 LISTENING TO THE GLOBAL COMMUNITY

These themes were explored further during the second International Maternal Newborn Health Conference (IMNHC), held in Nairobi in March 2026. Established in 2023, the IMNHC was created as a global platform for advancing maternal and newborn health through collaboration across research, policy, implementation, and advocacy. The Nairobi meeting brought together more than 1800 participants from over 100 countries, including clinicians, researchers, government representatives, civil society organizations, multilateral agencies, and public health leaders.

As part of the meeting, The Lancet Commission on Maternal and Newborn Health convened an interactive scientific session aimed not only at presenting ideas but also at listening to the global maternal and newborn health community. Through polling (*n* = 146 individual responses) and open discussion, participants shared perspectives on why progress has stalled, where current strategies are failing, and what priorities should define the next decade.

The responses revealed a striking consistency across regions and professional groups. Political commitment emerged as the single most important barrier to progress viewed by participants as even more critical than financing constraints, workforce shortages, or technical limitations. This finding echoes concerns raised decades ago by former FIGO President Mahmoud Fathalla, who famously stated that women die “not because we cannot act, but because we have yet to decide that their lives are worth saving.”

More than 30 years later, participants at the IMNCH meeting in Nairobi appeared to deliver a remarkably similar message. The challenge is not primarily the lack of knowledge about what works. Effective interventions for the major causes of maternal and newborn deaths including hemorrhage, hypertensive disorders, infection, preterm birth, and intrapartum complications have existed for years, and in many cases decades. The central challenge is implementing these interventions equitably, consistently, and at scale.

Participants also highlighted major concerns regarding the quality of care. In many settings, underuse and overuse of interventions coexist within the same health systems. While millions of women still lack access to life‐saving services, others are exposed to unnecessary medical interventions, including caesarean section rates exceeding 70% in some contexts. Participants emphasized that future progress cannot be measured solely by whether women reach healthcare facilities, but by whether the care they receive is safe, respectful, evidence‐based, and responsive to their needs and dignity.

A third major theme concerned data systems and accountability. Participants consistently emphasized that the problem is not simply a lack of data, but the failure to use data effectively. Too often, information systems serve global reporting requirements while offering little practical value for frontline providers, district health managers, or communities themselves. Participants called for simpler, more actionable data systems that strengthen local ownership, real‐time decision‐making, and meaningful accountability.

## 3 FROM EVIDENCE TO ACTION

Taken together, the discussions in Nairobi point toward a broader rethinking of maternal and newborn health in the years beyond the SDGs. They suggest that future progress will depend not only on technical innovation but also on confronting the political, institutional, and systemic factors that continue to prevent existing knowledge from being translated into action.

For The Lancet Commission on Maternal and Newborn Health, these perspectives provide both validation and direction. We will reinforce the importance of moving beyond narrow coverage indicators toward a broader understanding of health‐system performance, equity, quality, and women's lived experiences of care. We will also highlight the need to connect scientific evidence more directly to political accountability, governance, financing, and implementation.

The discussions in Nairobi did not reveal a lack of evidence. Rather, they revealed a persistent gap between what is known and what is done. Preventable maternal and newborn deaths continue not because solutions are unavailable, but because health systems, institutions, and political priorities too often fail to deliver those solutions equitably and consistently.

As the global health community moves beyond the SDG 2030 agenda, there is both an opportunity and an obligation to redefine what success in maternal and newborn health should mean in the decades ahead. The task facing The Lancet Commission is therefore not only to produce recommendations but also to help transform evidence into action that governments, institutions, and health systems can no longer afford to postpone.

*Pregnancy* warmly welcomes submissions addressing the global maternal health agenda as well as high‐quality international research across the full spectrum of pregnancy and maternal health. We encourage contributions using a wide range of methodological approaches, including clinical, epidemiological, translational, qualitative, and basic science research, covering all aspects of pregnancy, childbirth, and pregnancy outcomes.
